# ‘Virus Carriers’ and HIV testing: navigating Ukraine’s HIV policies and programming for female sex workers

**DOI:** 10.1186/s12961-019-0415-4

**Published:** 2019-02-28

**Authors:** Anna Tokar, Jacob Osborne, Kateryna Slobodianiuk, Dirk Essink, Jeffrey V. Lazarus, Jacqueline E. W. Broerse

**Affiliations:** 1 Barcelona Institute for Global Health (ISGlobal), Hospital Clínic - University of Barcelona, Rosselló 132, 2nd, ES-08036 Barcelona, Spain; 2 Athena Institute, Faculty of Science, Vrije Universiteit Amsterdam, De Boelelaan, 1085, NL-1081 HV Amsterdam, Netherlands; 3 Amsterdam Public Health Research Institute (APH), De Boelelaan, 1085, Amsterdam, NL-1081 HV Netherlands; 4 International Charitable Foundation Alliance for Public Health, 9th floor, building 10A, 5 Dilova, Kyiv, 03150 Ukraine

**Keywords:** Policy analysis, FSWs, HIV, Ukraine

## Abstract

**Background:**

There are an estimated 80,100 female sex workers (FSWs) in Ukraine, of whom 7% are living with HIV. Early HIV diagnosis continues to be a public health priority in Ukraine as only approximately 54% of people living with HIV are diagnosed nationwide. This study aims to analyse the content, context and discourse of HIV testing policies among female sex workers in Ukraine and how these policies are understood and implemented in practice.

**Methods:**

To analyse past and current national policies, we searched the database of the Ukrainian Parliament and the Ministry of Health for relevant policy documents (e.g. legislation and orders). To analyse the day-to-day practice of those involved in the implementation of these HIV programmes, we conducted face-to-face semi-structured interviews with key stakeholders. All data were coded using deductive thematic analysis initially guided by the Policy Triangle, a framework which addresses policy content, the process of policy-making, the health policy context, actors involved in policy formulation and implementation.

**Results:**

HIV testing policies are formed and implemented in the post-Soviet context through a vertical system of AIDS clinics, resulting in the separation of key affected populations from the rest of the health system. Successive testing policies have been strongly influenced by international donors and non-governmental organisations. Furthermore, a lack of government funding for HIV prevention created a gap that international donors and local non-governmental organisations covered to ensure the implementation of testing policies. Their role, however, had limited influence on the Ukrainian government to increase funding for prevention, including testing of FSWs. Since the early 1990s, when stigmatising and discriminatory forced/mandatory HIV testing was applied, these approaches were slowly replaced with voluntary testing, self-testing and assisted HIV testing, yet stigma was found to be a barrier among FSWs to access testing.

**Conclusion:**

Poor governance and the fragmentation of the health system, ongoing health sector reforms, shrinking international funding, and persisting stigma towards people living with HIV and sex workers might impede the continuity and sustainability of HIV testing programmes. Local civil society may now have the opportunity to contribute to the development and further implementation of HIV testing policies in Ukraine.

**Electronic supplementary material:**

The online version of this article (10.1186/s12961-019-0415-4) contains supplementary material, which is available to authorized users.

## Background

Initially fuelled by unsafe injection drug use, the HIV and AIDS epidemic in Ukraine remains one of the most severe in the WHO European Region, with current estimates of HIV prevalence in the general population at 0.99% [1]. At the beginning of 2017, 127,620 people living with HIV were officially enrolled in care at the Ukrainian AIDS clinics, which constituted up to 54% of the estimated population living with HIV [1, 2]. Since 2008, HIV incidence among the general population has slightly declined and the reported predominant attributed route of HIV transmission shifted from unsafe drug injecting to unprotected heterosexual contact [3–5]. HIV prevalence is disproportionally higher among key affected populations, including female sex workers (FSWs), people who inject drugs and men who have sex with men [3, 6]. Currently, there are an estimated 80,100 FSWs in Ukraine [7], approximately 7% of whom are living with HIV [8]. It is not known how many FSWs are enrolled in HIV care, as this type of data is not collected in Ukraine.

The Ukrainian national HIV prevention campaign officially started in 1991 when the first law to fight the HIV/AIDS epidemic was initiated by the Ukrainian Parliament (*Verkhovna Rada*). However, since the first National Guidelines on voluntary HIV testing and counselling were issued 14 years later [9], timely diagnosis has remained a problem among key affected populations, including FSWs. Over the years, there has been little attention paid to the context of HIV testing policy and its formulation. Therefore, we argue that a comprehensive examination of HIV policies, their discourse and the ways that various actors participate in their creation and implementation would help to understand how the policy context was created and how it evolved, as well as reveal gaps and discuss potential ways for improvement.

In this paper, we aim to analyse the content and context of HIV (testing) policies with a focus on FSWs as well as to describe how these policies are understood and implemented in practice. There is a need for more knowledge on the connections between the policies, the organisations that implement them and the individuals that may benefit (or not) from their implementation in order to inform future policies and practices. We prioritise the early diagnosis of FSWs due to the increased number of reported heterosexual transmissions in the country and numerous and concurrent intersections with networks of people who inject drugs [1, 4].

## Methods

### Data collection

**Fig. 1:**
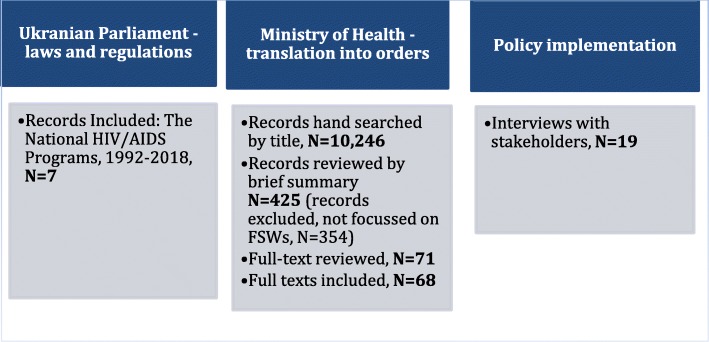
Flow chart of data collection

In order to examine how the HIV/AIDS programme was conceptualised, we used a framework analysis initially developed by Goffman et al. [10] and adapted by Caldwell et al. [11]. Using a variant of Goffman’s frame analysis, this model seeks to examine the pace, directions and impact of organisational innovations and change across different policy-making levels. We modified definitions of macro, meso and micro levels developed by Caldwell et al. [10] to fit the policy context of Ukraine. Thus, we started by identifying and analysing laws issued by the Ukrainian Parliament, and then examined how laws were introduced through orders issued by the Ministry of Health of Ukraine. The day-to-day practice of individuals working at the implementation level and their understanding of the framework were subsequently investigated through face-to-face semi-structured interviews*.* The process of data collection is described in Fig. 1.

### Database analysis

The Constitution of Ukraine sets out several state bodies that can issue health policies with varying degrees of power, including the Parliament of Ukraine, Cabinet of Ministries of Ukraine, President, Ministry of Health of Ukraine and State Committees. The Parliament is the sole legislative body in Ukraine; the laws issued should determine all other health policies. All other state bodies issue health policies (e.g. regulations, orders and recommendations) of equal legal power and with no direct hierarchy among them. For the purpose of the current study, we systematically collected policies issued by the Parliament (as the highest legislative body) and by the Ministry of Health (as the highest body of healthcare system). Policies of other bodies were not collected systematically, although some were used in the discussion. We collected National Programmes of HIV/AIDS in Ukraine, which have had the status of a national law since 2009 (prior to that, the National Programme of HIV/AIDS had the status of a decree of the Cabinet of Ministers of Ukraine). Since 1992, seven policies have been issued. At the level of the Ministry of Health, the first author (AT) hand-screened titles of 10,246 documents issued by the Ministry of Health of Ukraine during the 2006–2015 period. We collected 544 policies with terms that covered the terms ‘HIV’ and ‘test’; we used terms translated into Ukrainian (Additional file 1). Next, all citations were exported into an Excel database and brief summaries were screened. The inclusion criteria were (1) publication between January 2006 and December 2015, and (2) presenting data on HIV testing of FSWs. We excluded duplicates and all citations focused on testing of blood donors and pregnant women (*n* = 464). After reading the full text of 77 records of pre-accepted citations, we collected 68 papers for analysis at all three levels (Table 1).

**Table 1:** HIV policies of Ukraine, 1992–2015

*N*	Title	Type of document	Edition	Year	Targeted populations
1	National programme to fight HIV/AIDS in Ukraine, 2014–2018	National law, issued by *Verkhovna Rada* of Ukraine	First edition	20.11.2014 [12]	General population, including key affected populations
2	National programme to fight HIV/AIDS in Ukraine, 2009–2013	National law, issued by *Verkhovna Rada* of Ukraine	First edition	19.02.2009 [13]	General population, including key affected populations
3	National programme to fight HIV/AIDS in Ukraine, 2004–2008	National law, issued by *Verkhovna Rada* of Ukraine	First edition	04.03.2004 [14]	General population, including key affected populations
4	National programme to fight HIV/AIDS in Ukraine, 2001–2003	National law, issued by *Verkhovna Rada* of Ukraine	First edition	11.07.2001 [15]	General population, including key affected populations
5	National programme to fight HIV/AIDS in Ukraine, 1999–2000	National law, issued by *Verkhovna Rada* of Ukraine	First edition	09.03.1999 [16]	General population, including key affected populations
6	National programme to fight HIV/AIDS in Ukraine, 1995–1998	National law, issued by *Verkhovna Rada* of Ukraine	First edition	14.03.1995 [17]	General population, including key affected populations
7	National programme to fight HIV/AIDS in Ukraine, 1992–1994	National law, issued by *Verkhovna Rada* of Ukraine	First edition	27.02.1992 [18]	General population, including key affected populations
8	About the distribution of test systems for rapid diagnosis of antibodies to HIV-1 and -2 and diagnosis of opportunistic infections amongst people with HIV/AIDS, which were bought out of the costs of the National Budget of Ukraine, 2005	National Order, Issued by the Ministry of Health of Ukraine	First edition	03.02.2006 No. 42 [19]	General population, including key affected populations
9	About the distribution of reagents for haematological diagnostics, which were provided by the International HIV/AIDS Alliance in Ukraine as humanitarian aid according to the Donation Agreement, 22 April, 2005	National Order, Issued by the Ministry of Health of Ukraine	First edition	07.02.2006 No. 59 [20]	General population, including key affected populations
10	About the approval of the registration form No. 498–2/о ‘Result of immunochromatographic diagnostic’ (CITO TEST)	National Order, Issued by the Ministry of Health of Ukraine	First edition	24.03.2006 No. 158 [21]	General population, including key affected populations
11	About the establishment of the Reference laboratory for HIV/AIDS diagnosis under there regulation of the Ukrainian AIDS Center of Ministry of Health of Ukraine	National Order, Issued by the Ministry of Health of Ukraine	First edition	17.04.2006 No. 230 [22]	General population, including key affected populations
12	About the implementation of the order of the voluntary counselling and testing for HIV (the protocol) in the health facilities	National Order, Issued by the Ministry of Health of Ukraine	First edition	19.04.2006 No. 236 [23]	General population, including key affected populations
13	About the approval of the typical regulations of the *Dovira* cabinet	National Order, Issued by the Ministry of Health of Ukraine	First edition	27.06.2006 No. 421 [24]	General population, including key affected populations
14	About the approval of the Instruction to implement the regulations of the VCT (the protocol) in the TB centres, STI clinics and addiction clinics	National Order, Issued by the Ministry of Health of Ukraine	First edition	06.07.2006 No. 446 [25]	General population, including key affected populations
15	About the distribution of test systems for conformational diagnosis of HIV, which were bought out of the costs of the National Budget of Ukraine, 2006	National Order, Issued by the Ministry of Health of Ukraine	First edition	01.11.2006 No. 722 [26]	General population, including key affected populations
16	About the distribution of test systems for HIV diagnosis among patients of TB and STI clinics	National Order, Issued by the Ministry of Health of Ukraine	First edition	21.12.2006 No. 850 [27]	General population, including key affected populations
17	About the modification of the order of Ministry of Health of Ukraine, 01.11.06 No. 72	National Order, Issued by the Ministry of Health of Ukraine	Modification	29.01.2007 No. 39 [28]	General population, including key affected populations
18	About the approval of the Comprehensive plan of activities to expand express testing for HIV in Ukraine in 2007	National Order, Issued by the Ministry of Health of Ukraine	First edition	11.04.2007 No. 179 [29]	General population, including key affected populations
19	About the approval of the primary registration forms of the usage of rapid tests in health facilities and their completion	National Order, Issued by the Ministry of Health of Ukraine	First edition	06.06.2007 No. 304 [30]	General population, including key affected populations
20	Modification of order issued on 28.12.2005 No. 773 About the distribution of test systems and reagents for HIV diagnosis amongst patients of TB and STI clinics, which were bought out of the costs of the National Budget of Ukraine, 2005	National Order, Issued by the Ministry of Health of Ukraine	Modification	03.07.2007 No. 366 [31]	General population, including key affected populations
21	About the distribution of rapid test systems (express) for diagnosis of HIV-1 and -2, which were bought out of the costs of the National Budget of Ukraine, 2007	National Order, Issued by the Ministry of Health of Ukraine	First edition	05.09.2007 No. 530 [32]	General population, including key affected populations
22	Order issued on 16.11.2007 No. 731 about modification of the order issued 05.09.07 No. 530	National Order, Issued by the Ministry of Health of Ukraine	Modification	16.11.2007 No. 731 [33]	General population, including key affected populations
23	About the distribution of reagents and control serums for biochemical diagnostics, which were bought out of the costs of the National Budget of Ukraine, 2007	National Order, Issued by the Ministry of Health of Ukraine	First edition	12.12.2007 No. 821 [34]	General population, including key affected populations
24	About functioning of the *Dovira* cabinet	National Order, Issued by the Ministry of Health of Ukraine	First edition	25.02.2008 No. 102 [35]	General population, including key affected populations
25	About the approval of the working group to develop methodology for collection and calculation needs of regions in medicines, including test systems, equipment for PLWH	National Order, Issued by the Ministry of Health of Ukraine	First edition	28.03.2008 No. 102-Адм [36]	General population, including key affected populations
26	About the distribution of the reagents for diagnosis of the provirus DNA of HIV-1 by the International HIV/AIDS Alliance in Ukraine as a humanitarian aid in 2008	National Order, Issued by the Ministry of Health of Ukraine	First edition	09.04.2008 No. 193 [37]	General population, including key affected populations
27	About the distribution of the test systems for conformational diagnosis by IFA; distribution of diagnostic test systems of antigen p24 of HIV-1 and for immunoblot	National Order, Issued by the Ministry of Health of Ukraine	First edition	04.07.2008 No. 354 [38]	General population, including key affected populations
28	About the implementation of a pilot project entitled Expanding access for most at risk populations towards HIV/AIDS to counselling and testing for HIV with rapid test during 2008–2009	National Order, Issued by the Ministry of Health of Ukraine	First edition	08.08.2008 No. 440 [39]	Key affected populations
29	About the quality assessment of test system for HIV diagnosis by IFA method (DIA-HIV-Ag/Ab, DIA-HIV-p24), which is produced by ATZT NVK Diaprom-Med, Ukraine	National Order, Issued by the Ministry of Health of Ukraine	First edition	27.10.2008 No. 612 [40]	General population, including key affected populations
30	About the distribution of reagents and control serums for biochemical diagnostics, which were bought out of the costs of the National Budget of Ukraine, 2008	National Order, Issued by the Ministry of Health of Ukraine	First edition	29.12.2008 No. 796 [41]	General population, including key affected populations
31	About modification of the order of Ministry of Health of Ukraine issued on 09.04.2008 No. 193	National Order, Issued by the Ministry of Health of Ukraine	Modification	13.03.2009 No. 164 [42]	General population, including key affected populations
32	About modification of the order of Ministry of Health of Ukraine issued on 04.07.2008 No. 354	National Order, Issued by the Ministry of Health of Ukraine	Modification	22.04.2009 No. 270 [43]	General population, including key affected populations
33	About modification of the order of Ministry of Health of Ukraine issued on 04.07.2008 No. 354	National Order, Issued by the Ministry of Health of Ukraine	Modification	24.04.2009 No. 273 [44]	General population, including key affected populations
34	About the approval of plan of activities of the National Programme to fight HIV/AIDS 2009–2013	National Order, Issued by the Ministry of Health of Ukraine	First edition	25.06.2009 No. 452 [45]	General population, including key affected populations
35	About the approval of Strategy of the enhancement of HIV counselling and testing, and testing standardised laboratory diagnostics 2009	National Order, Issued by the Ministry of Health of Ukraine	First edition	14.07.2009 No. 509 [46]	General population, including key affected populations
36	About the modification of the order of Ministry of Health of Ukraine, 04.07.2008 No. 354	National Order, Issued by the Ministry of Health of Ukraine	Modification	27.07.2009 No. 536 [47]	General population, including key affected populations
37	About the distribution of test systems for conformational diagnosis of HIV by detection of antigen p24 HIV-1 and immunoblot, which were bought out of the costs of the National Budget of Ukraine, 2009	National Order, Issued by the Ministry of Health of Ukraine	First edition	18.08.2009 No. 611 [48]	General population, including key affected populations
38	About the distribution of test systems for diagnosis of provirus DNA of HIV-1, which were bought out of the costs of the National Budget of Ukraine, 2009	National Order, Issued by the Ministry of Health of Ukraine	First edition	18.08.2009 No. 612 [49]	General population, including key affected populations
39	About the approval of the temporary regulations of the diagnosis of HIV with rapid tests, their usage and registration of test results	National Order, Issued by the Ministry of Health of Ukraine	First edition	27.08.2009 No. 578 [50]	General population, including key affected populations
40	About the modification of the order of Ministry of Health of Ukraine, 04.07.2008 No. 354	National Order, Issued by the Ministry of Health of Ukraine	Modification	27.08.2009 No. 642 [51]	General population, including key affected populations
41	About the distribution of test systems for conformational diagnosis of HIV-1 and -2, which were bought out of the costs of the National Budget of Ukraine, 2009	National Order, Issued by the Ministry of Health of Ukraine	First edition	15.12.2009 No. 951 [52]	General population, including key affected populations
42	About the distribution of test systems for conformational diagnosis of HIV by IFA, which were bought out of the costs of the National Budget of Ukraine, 2009	National Order, Issued by the Ministry of Health of Ukraine	First edition	15.12.2009 No. 953 [53]	General population, including key affected populations
43	About the modification of the order of Ministry of Health of Ukraine, 18.08.2009 No. 611	National Order, Issued by the Ministry of Health of Ukraine	Modification	12.02.2010 No. 103 [54]	General population, including key affected populations
44	About the distribution of test systems for HIV diagnosis amongst most at risk populations, which were bought by the Global Fund to fight HIV/AIDS, TB and Malaria in 2010	National Order, Issued by the Ministry of Health of Ukraine	First edition	08.04.2010 No. 316 [55]	Key affected populations
45	About the modification of the order of Ministry of Health of Ukraine, 18.08.2009 No. 612	National Order, Issued by the Ministry of Health of Ukraine	Modification	19.04.2010 No. 344 [56]	General population, including key affected populations
46	About the distribution of reagents for biochemical diagnostics for PLWH, which were bought out of the costs of the National Budget of Ukraine, 2010	National Order, Issued by the Ministry of Health of Ukraine	First edition	29.09.2010 No. 828 [57]	General population, including key affected populations
47	About the distribution of test systems for conformational diagnosis of antibodies to HIV-1 and -2 by IFA and test systems to detect antigen р24 HIV-1	National Order, Issued by the Ministry of Health of Ukraine	First edition	20.10.2010 No. 893 [58]	General population, including key affected populations
48	About the distribution of the rapid test systems (express) for diagnosis of antibodies to HIV-1 and -2, which were bought out of the costs of the National Budget of Ukraine, 2010	National Order, Issued by the Ministry of Health of Ukraine	First edition	20.10.2010 No. 898 [59]	General population, including key affected populations
49	About the approval of the Regulations of HIV diagnosis and quality control of diagnosis, the primary registration forms and instruction of their completion	National Order, Issued by the Ministry of Health of Ukraine	First edition	21.12.2010 No. 1141 [60]	General population, including key affected populations
50	About the modification of the order of Ministry of Health of Ukraine, 20.10.2010 No. 893	National Order, Issued by the Ministry of Health of Ukraine	Modification	17.01.2011 No. 8 [61]	General population, including key affected populations
51	About the modification of the order of Ministry of Health of Ukraine, 20.10.2010 No. 893	National Order, Issued by the Ministry of Health of Ukraine	Modification	27.07.2011 No. 437 [62]	General population, including key affected populations
52	About the modification of the order of Ministry of Health of Ukraine, 20.10.2010 No. 893	National Order, Issued by the Ministry of Health of Ukraine	Modification	30.08.2011 No. 552 [63]	General population, including key affected populations
53	About the distribution of reagents for diagnosis of provirus DNA HIV-1 in the format of the ‘real time’, which were bought out of the costs of the National Budget of Ukraine, 2011	National Order, Issued by the Ministry of Health of Ukraine	First edition	05.12.2011 No. 864 [64]	General population, including key affected populations
54	About losing eligibility of the order of the Ministry of Health, issued on 22.02.2002 No. 71	National Order, Issued by the Ministry of Health of Ukraine	Modification	05.12.2011 No. 866 [65]	General population, including key affected populations
55	About the distribution of test systems for conformational diagnosis of HIV by detection antibodies to HIV by immunoblot and IFA and detection of antigen p24 HIV-1, which were bought out of the costs of the National Budget of Ukraine, 2011	National Order, Issued by the Ministry of Health of Ukraine	First edition	05.12.2011 No. 875 [66]	General population, including key affected populations
56	About the distribution of test systems for diagnosis multi-infections amongst most at risk populations, which were bought by the Global Fund to fight HIV/AIDS, TB and Malaria for 2011	National Order, Issued by the Ministry of Health of Ukraine	First edition	03.02.2012 No. 85 [67]	Key affected populations
57	About the distribution of test systems for conformational diagnosis of HIV by detection antibodies to HIV by immunoblot and IFA, which were bought out of the costs of the National Budget of Ukraine, 2011	National Order, Issued by the Ministry of Health of Ukraine	First edition	09.02.2012 No. 102 [68]	General population, including key affected populations
58	About organisation of diagnostic services for testing for HIV, HBV, HCV and STIs in mobile clinics	National Order, Issued by the Ministry of Health of Ukraine	First edition	14.02.2012 No. 114 [69]	General population, including key affected populations
59	About the modification of the order of Ministry of Health of Ukraine, 05.12.2011 No. 875	National Order, Issued by the Ministry of Health of Ukraine	Modification	26.03.2012 No. 195 [70]	General population, including key affected populations
60	About the modification of the order of Ministry of Health of Ukraine, 05.12.2011 No. 864	National Order, Issued by the Ministry of Health of Ukraine	Modification	25.04.2012 No. 309 [71]	General population, including key affected populations
61	About the modification of the order issued on 5 of December of 2011, No. 875 About the distribution of test systems for conformational diagnosis of HIV by detection antibodies to HIV by immunoblot and IFA, which were bought out of the costs of the National Budget of Ukraine, 2011 (with changes)	National Order, Issued by the Ministry of Health of Ukraine	Modification	04.09.2012 No. 684 [72]	General population, including key affected populations
62	About the distribution of reagents for diagnosis of provirus DNA HIV-1 in the format of the ‘real time’, which were bought out of the costs of the National Budget of Ukraine, 2012	National Order, Issued by the Ministry of Health of Ukraine	First edition	05.11.2012 No. 873 [73]	General population, including key affected populations
63	About the distribution of materials for diagnosis of provirus DNA HIV-1 in the format of the ‘real time’, which were bought out of the costs of the National Budget of Ukraine, 2012	National Order, Issued by the Ministry of Health of Ukraine	First edition	12.11.2012 No. 910 [74]	General population, including key affected populations
64	About the distribution of reagents for internal quality control of HIV diagnostics, which were bought out of the costs of National Budget of Ukraine, 2012	National Order, Issued by the Ministry of Health of Ukraine	First edition	16.11.2012 No. 919 [75]	General population, including key affected populations
65	About the he distribution of the conformational diagnostics of antibodies to HIV by immunoblot and IFA and detection of the antigen р24 HIV-1 (conformational test system), which were bought out of the costs of the National Budget of Ukraine, 2012	National Order, Issued by the Ministry of Health of Ukraine	First edition	20.11.2012 No. 935 [76]	General population, including key affected populations
66	About the distribution of the IFA test systems for diagnostics of antigen р24 HIV-1 and -2, which were bought out of the costs of the National Budget of Ukraine, 2012	National Order, Issued by the Ministry of Health of Ukraine	First edition	21.12.2012 No. 1094 [77]	General population, including key affected populations
67	About the modification of the order of Ministry of Health of Ukraine, issued on 20.11.2012, No. 935	National Order, Issued by the Ministry of Health of Ukraine	Modification	18.05.2013 No. 395 [78]	General population, including key affected populations
68	About the distribution of materials and reagents for diagnosis of provirus DNA HIV-1 in the format of ‘real time’ by PCR, which were bought out of the costs of the National Budget of Ukraine, 2013	National Order, Issued by the Ministry of Health of Ukraine	First edition	17.06.2013 No. 512 [79]	General population, including key affected populations
69	About the distribution of reagents for internal quality control of HIV diagnostics, which were bought out of the costs of National Budget of Ukraine, 2013	National Order, Issued by the Ministry of Health of Ukraine	First edition	27.06.2013 No. 559 [80]	General population, including key affected populations
70	About the distribution of the test systems for conformational diagnostics by IFA and immunological diagnostics of HIV-1 by immunoblot, which were bought out of costs of National budget of Ukraine, 2013	National Order, Issued by the Ministry of Health of Ukraine	First edition	08.08.2013 No. 711 [81]	General population, including key affected populations
71	About the modification of the order of Ministry of Health of Ukraine, 17.06.2013 No. 512	National Order, Issued by the Ministry of Health of Ukraine	Modification	21.10.2013 No. 896 [82]	General population, including key affected populations
72	About the distribution of the test systems for diagnostics of antigen p24 HIV-1 by IFA, which were bought out of costs of National Budget of Ukraine, 2013	National Order, Issued by the Ministry of Health of Ukraine	First edition	21.10.2013 No. 897 [83]	General population, including key affected populations
73	About the modification of the order of Ministry of Health of Ukraine, 08.08.2013 No. 711	National Order, Issued by the Ministry of Health of Ukraine	Modification	16.05.2014 No. 336 [84]	General population, including key affected populations
74	About the modification of the order of Ministry of Health of Ukraine, 21.10.2013 No. 897	National Order, Issued by the Ministry of Health of Ukraine	Modification	02.06.2014 No. 377 [85]	General population, including key affected populations
75	About the modification of the order of Ministry of Health of Ukraine, 08.08.2013 No. 711	National Order, Issued by the Ministry of Health of Ukraine	Modification	17.02.2015 No. 72 [86]	General population, including key affected populations

*FRT* real-time hybridization-fluorescence detection of amplified products, *IFA* immunofluorescence assay, *PLWH* people living with HIV, *STIs* sexually transmitted infections, *TB* tuberculosis, *VCT* voluntary counselling and testing

The third author (KS) independently screened titles and abstracts of 10% of the selected policy documents to check that all retained documents met the inclusion criteria. Both authors showed a high agreement rate (91%), with all discrepancies resolved.

### Semi-structured interviews

To examine the views and perceptions of stakeholders towards HIV testing policies among FSWs in Ukraine, we conducted 19 semi-structured interviews (18 face-to-face and one via Skype) with individuals who are directly involved in or affected by HIV policies in relation to testing of FSWs during April–June 2017 (Box 1). We identified key stakeholders in discussions with our local partner (Alliance for Public Health) and through snowballing (every interviewee was asked to suggest other relevant stakeholders). We did not examine how HIV policies are developed or implemented in the war zone (Donetsk and Lugansk) and Crimea; yet, to gain some understanding of how war affects HIV testing, we conducted one interview with the director of a non-governmental organisation (NGO) working with FSWs in Donetsk. Invitations were sent by e-mail, describing in brief the purpose of the study and contact information. The interview guide can be found in Additional file 2. The duration of the interviews was approximately 90 min and no remuneration was provided. Interviews were conducted either in English (*n* = 15), Russian or Ukrainian (*n* = 4). Non-native English-speaking interviewees were proposed to use Ukrainian or Russian languages. All interviews were digitally recorded and transcribed. Those interviews held in Russian and Ukrainian were subsequently translated into English. The two bilingual authors (AT and KS) agreed on the final translations. Next, all interviews were imported into ATLAS.ti.

### Data analysis

**Fig. 2:**
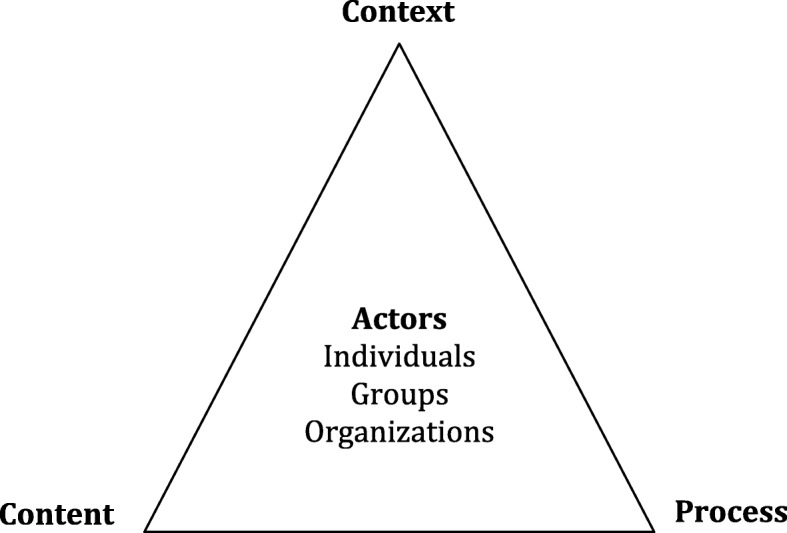
Policy triangle [88]

Data was coded using deductive thematic coding analysis [87], initially guided by the Policy Triangle [88], a framework which addresses policy content, the process of policy-making, the context of health policy as well as actors involved in policy formulation and implementation, including individuals, groups and organisations (Fig. 2).

Two researchers (AT and JO) independently read and coded data. Mid-range theories were developed jointly by the research team members as we grouped individual codes into categories and then merged them in conceptually coherent themes (thematic coding analysis). Further, two researchers (AT and JO) re-visited all the data applying the coding framework; if discrepancies arose, they were resolved through discussions with a third researcher (DE). The final coding framework of database analysis and interviews slightly differed as interviews allowed us to examine a stakeholder’s relationship dynamic, process of policy delivery and ideas on the future of HIV/AIDS programmes in Ukraine as well as to address the influence of the ongoing war in the east of the country on the AIDS epidemic among FSWs, including HIV testing (Additional file 1).

We used critical discourse analysis, which aims to explore relationships between language-in-use (practices, events and texts) and the broader social and cultural context (processes, relations and structures) [89, 90], to trace how HIV-testing policies were constructed and how they changed over time in their historical context. Although we use critical discourse analysis to illustrate how texts shape the representation of the world, social relationships and social identities, we analysed the content of HIV-testing polices in relation to key affected populations, including FSWs, in order to reflect on how power, justice and equity were addressed and if there were any changes over time.

### Ethics

Ethical approval was obtained from the Ukrainian Institute on Public Health Policy (No. 21/IRB, 20.02.2017). Verbal informed consent was obtained before each interview; no personal information was recorded. The content of the interview and the identity of the interviewee were kept anonymous in the discussion of the study results.

## Results

### Policies related to HIV testing: content

#### Laws and regulations

In the early 1990s, national HIV/AIDS programmes focused on the forced testing of those ‘suspected to have HIV’, controlling migration flows with force (involving the police to detect those who should be tested and not on an individual voluntary basis, but testing of all immigrants, sailors, professional athletes and everyone travelling abroad). During the period from 1987 to 1994, tests were carried out among a long list of populations, including blood donors, pregnant women, sexual partners of people living with HIV, professional contacts with people living with HIV, hospitalised patients, military personnel, people who were abroad for more than 3 months, ‘promiscuous persons’, patients with sexually transmitted infections (STIs), prisoners, men who have sex with men, and drug addicts. All these efforts (39 million tests) resulted in only 398 positive test results [5]. In 1994, the testing strategy therefore shifted from mass to ‘forced’ and, after 2000, to mandatory testing with large-scale testing among the key affected populations, including FSWs. Key affected populations were negatively represented as those responsible for spreading AIDS and for bringing it into the country initially:

“*The presence of a significant number of HIV-infected narcomaniacs in the countries bordering with Ukraine (Poland, Hungary, Slovakia, Belarus) have created conditions for the spread of HIV/AIDS among narcomaniacs in Ukraine.*” (National HIV/AIDS Programme, 1992–1994) [12]

For that reason, separate facilities (AIDS clinics) for people living with AIDS and ‘HIV carriers’ were established:

“*To achieve the proper level of organization medical and preventive care for people with AIDS and HIV carriers it is necessary to determine the annual regulatory requirements for: medical personnel needed to maintain care of patients and infection carriers for the period until 1994 year, and to determine approximate needs in premises and equipment for specialized departments of hospitals.*” (National HIV/AIDS Programme, 1992–1994) [12]

For almost a decade, until the early 2000s, key affected populations were blamed and victimised in legislation, their human rights violated through forced and later mandatory testing in order to guarantee the “*social protection of citizens*” and “*healthy people*” (Table 2):

“*To guarantee purposeful work of the employees of Internal Affairs office in order to identify persons belonging to risk groups – narcomaniacs, prostitutes and vagrants, as well as cults of debauchery, etc. Submit timely to the health authorities appropriate information for mandatory medical examination of these persons for the purpose of detection of virus carriers and diseased with AIDS* […] *when investigating and prosecuting sexual crimes about transmitting sexually transmitted diseases and avoidance of treatment to conduct medical examinations, to take measures to provide examinations of AIDS criminals. To strengthen prosecutorial oversight of the investigation of such cases.*” (National HIV/AIDS Programme, 1992–1994) [12]

“*In order to improve the legal framework of the prevention of drug addiction and humanization in the field of treatment and rehabilitation of patients for drug addiction to develop and submit propositions on expediency of the abolition of forced treatment of patients with addiction and replacement it for mandatory to be considered by the National Coordination Council for Combating Addiction under the Cabinet of Ministers Ukraine.*” (National HIV/AIDS Programme, 1999–2000) [14]

**Table 2:** Word counts of terms related to key affected populations: HIV policies of Ukraine, 1992–2015

Word counts, number of times used	1992–1994	1995–1998	1999–2000	2001–2003	2004–2008	2009–2013	2014–2018
National Programme, years							
Diseased with AIDS	31	23	17	6	44	20	6
Diseased with narcomania			12				
Groups of increased risk							10
Groups of people with behaviour not approved by society		1					
HIV infected		35		18	41	22	3
Homosexuals/homosexual contact	2	1					
Injecting drug users/Injection drug use				7	7	3	
Injecting narcomaniacs			2				
Injecting opioid drug users							1
Narcomania		4	42				
Narcomaniacs	3	6	5	1			
Narcomaniacs law breakers			1				
Cults of debauchery	1						
Persons who are engaged in prostitution						1	
People living with HIV							16
Representatives of the risk groups						5	
People providing sexual services for payment				2			
People suspected to have HIV	1		13				
People who belong to at-risk groups	4	4					
People who inject drugs		5					
People who suffered because of HIV					3		
Prostitutes/prostitution	2			1	1		
Prostitutes who are identified as such according to the law		1					
Sources of HIV		1					
Virus/HIV carriers	17	1					
Vulnerable groups				9	19		

Nevertheless, we found that, within every new National HIV/AIDS Programme, the discourse became less stigmatising and more inclusive, referring to the international evidence and experience, calling for “*international collaboration*” and “*extending partnership*” as well as recognising the role of key affected populations. Consequently, the representation of civil society, including NGOs, as a powerful actor in policy development of HIV-testing policies (through working groups) and policy implementation, also increased over time. According to some interviewees, these changes in the representation and role of key affected populations might be partly attributed to the presence and efforts of international donors (including the Global Fund, WHO, USAID, and the Open Society Foundation), whose financial support had the power to push for human rights, greater equality and inclusiveness for key affected populations.

#### Translation into orders

We examined 68 national orders on HIV testing issued by the Ministry of Health of Ukraine from the years 2006 to 2015 to study how policies are translated into working structures to be implemented on the ground. Most orders were issued to allocate and distribute resources across AIDS clinics, including test systems, test equipment and other supplies; only 12 orders were issued either to introduce new healthcare structures and their functioning or to provide an extensive description of new approaches in testing (e.g. rapid testing, voluntary counselling and testing). These orders illustrate how the HIV-testing programme was developed in Ukraine, among which are the following:

- Establishment of the Reference Laboratory – orders Nos. 230 and 236, 2006 [22, 23];
- Introduction of voluntary counselling and testing – order Nos. 236 and 446, 2006 [23, 25];
- Introduction of rapid (express) testing – order No. 304, 2007 [30];
- Approval of comprehensive plan of activities to expand express HIV testing – order No. 179, 2007 [29];
- Establishment of the *Dovira* cabinets (small testing sites to provide testing available in the rural areas, where there are no AIDS clinics) – orders: No. 421, 2006, and No. 102, 2008 [24, 25];
- Establishment of mobile clinics – order No. 114, 2012 [69].

The central government body, the Ministry of Health of Ukraine, mediates and controls the movement of every single unit of products at the regional level:

“*… according to the request of the chief physician of the Zaporizhzhya Regional AIDS Center to redistribute 15 sets of test systems New Law Blot 1 and request of the First Deputy Minister of Crimea with a request to provide 15 sets of test systems New Law Blot…*” (The Order of The Ministry of Health of Ukraine No. 39, 2007) [28]

In order to maintain this system, approximately 1000 orders have been issued by the Ministry of Health each year (926 in 2006; 890 in 2007; 833 in 2008; 1084 in 2009; 1203 in 2010; 1028 in 2011; 1141 in 2012; 1169 in 2013; 1039 in 2014; 933 in 2015).

### Policy actors

Overall, policy actors included three major groups, namely government agencies, including the National Parliament (*Verkhovna Rada*) and the Ministry of Health with regional subdivisions, AIDS clinics, hospitals, *Dovira* cabinets and research agencies, as well as civil society organisations (CSOs) and international donors.

Government agencies, particularly the National Parliament and the Ministry of Health, have been key policy actors in setting the national policies on HIV/AIDS in general and HIV testing in particular. However, all 19 respondents also mentioned the importance of civil society in Ukraine in implementing HIV policies and inspiring national policies. Furthermore, CSOs and HIV/AIDS programmes are generally funded by international donors such as the Global Fund. The result has been a focused response to HIV, but one that largely runs parallel to the overall health system in the country. In general, local NGOs like the International Alliance for Public Health or All-Ukrainian Network of People Living with HIV first developed and implemented innovative HIV testing approaches in Ukraine with support of international donors, which were then adopted by the government and officially translated into national policies. For example, the first HIV testing programme through mobile clinics was implemented in Odessa in 2004 by local NGOs, at a time when no national HIV policies on mobile clinics were in place. The national HIV testing policies dealing with mobile clinics were issued 8 years later (order No. 114, 2012). Similarly, in 2006, the Alliance for Public Health in Ukraine launched the first HIV testing intervention with rapid diagnostic tests across 12 pilot sites as part of a comprehensive harm-reduction programme for key affected populations. The intervention was subsequently scaled up nationwide in 2007 and by 2014 had been implemented in 25 *oblasts* (administrative regions) [91, 92]. Official policy on how to conduct rapid HIV testing was issued in 2007 (order No. 304).

### Policy context

#### Soviet legacy

Within the present post-Soviet context, we identified several topics affecting policy development and implementation, including moral models leading to the victimisation of sex workers and apparent sympathy (“*we make these decisions for their own best interest*”) [89]. Thus, decisions to implement forced and, later, mandatory testing among sex workers were based on the moral models of the Soviet period, which limited sexuality and placed a focus on the so-called moral upbringing of youth, propaganda about ‘proper behaviour’ and the core role of the family. Even though some key affected populations can now voice their needs through several government and international platforms, it seems that their power is rather restricted by a ‘moral code’ in Ukrainian society. This is well illustrated by government acceptance to fund treatment for key affected populations, and its limited motivation with regard to prevention activities:

“*There was no such advocacy for it, if compared with treatment, the government is not used to give money for that… I think they don’t have the reflex to fund prevention.* [...] *this involves some inheritance of the old system, Soviet system and stigma and socio-economic environment. When we talk about it, we need to remember that politicians will remember about their voter*.” (P13, Donor)

In addition, a number of laws in Ukraine, including administrative (Article 181/1) and criminal (Articles 130, 302, 303) laws, either prohibit sex work directly or through subtler means such as the prosecution of purposefully spreading sexually transmitted infections, including HIV.

#### Health reform

The reform of the health system was taking place, or soon to take place, which involves replacing the post-Soviet healthcare model with a new one, yet to be developed. The initial stage of the reform is introducing a new concept for Ukraine – family doctors:

“*Right now, the health system reform is going on and they want to do the primary healthcare totally reformed in terms of financing. So, there will be family doctors who will be doing this primary healthcare. And they will get paid by government for each person. So, it’s like per capita system.*” (P06, NGO)

According to the interviewees, the goal of the successive stages is to introduce more government funding into HIV programmes progressively, a goal which they believe to be positive and will encourage sustainability and integration into the rest of the health system. It is expected that the government would directly pay NGOs through a system of ‘social contracting’ and ultimately replace all donor funding, including that from the Global Fund. In the meantime, several interviewees, including one who works for an international health agency, mentioned social contracting as a way of ensuring that most of the work that NGOs currently do continues to be done. In social contracting, regional governments are essentially hiring existing CSOs to work as they normally would, but with increased government funding and regulation. The representative from an international health agency highlighted how this contracting could assist in the transition to this goal of a more government-led HIV landscape and an integrated health system:

“*I would say that the social contracting should start sooner or later because the system will not be able to go and search for clients and I believe more in this than to have general testing by a primary care physician who just does millions of tests.*” (P16, Donor)

#### War in eastern Ukraine

The war in the eastern part of the country was not a central feature of most interviews as the area was not within the jurisdiction of most organisations. The government does not officially give support to the regions that are not under Ukrainian control. A United States doctor working for a United States agency suggested that the war is a source of tension for the Global Fund:

“*Programming in the uncontrolled areas is difficult because there’s no way to monitor what’s being done over there, and it goes against the Ukrainian policy to not provide support to those over there. It is in one sense a distraction, but also just the true logistics of working out any support because the authorities have acted capriciously*.” (P09, Donor)

The issue of internally displaced persons is also associated with the war and, interestingly, it was sometimes the only way that some interviewees could link the content of our conversations with the war. The project manager working for an international NGO described some of the epidemiological implications of internally displaced persons in Ukraine.

“*We had some regions with IDPs* [internally displaced persons]*. It’s the only way somehow we work regarding those regions in the east because as I said in Kharkiv region, we had a lot of IDPs and some of them come to the project. In Kiev within a specific project for HIV-positive women, it’s quite a small project, but we can see that out of 150 women with HIV in Kiev, I think 20 or 30 of them are internally displaced. So, it’s a huge number for such a small number of clients.*” (P07, NGO)

### Policy implementation

All respondents mentioned the importance of civil society in Ukraine, given that organisations and programmes are generally funded by international donors such as the Global Fund. A product of this has been a more vertical response to HIV, which is implemented partly by the state and partly by NGOs. To make the system complementary, key donors asked to see all the activities funded by all the donors and government in each and every country application, and joint tuberculosis and HIV/AIDS Global Fund country applications have been developed. This has had effects on multiple parts of the overall system of care for HIV, including a separation of key affected populations from the general public in different facilities, a fragmentation of services available to FSWs and others who use the HIV system of care, and a lack of priority given to prevention services.

#### Lack of resources

The Ukrainian health system is traditionally underfunded, with a total of 80% of funding allocated to hospital care, 15% for outpatient services, and only 5% for primary care and prevention. This created a gap, which has been filled by international funding. For example, since 2003, the biggest donor in the country, the Global Fund, has signed over US$555 million and disbursed over US$547 million in Ukraine [93].

Thus, over the years, international donors have funded HIV testing programmes among FSWs in Ukraine in various ways, including purchasing test systems, laboratory equipment and supplying materials; conducting training for health professionals and social workers; and developing and printing guidelines as well as direct funding of HIV testing. The National HIV/AIDS Programme clearly defines government and donor roles, responsibilities and the funds needed to implement them. International donors (e.g. the Global Fund) have financed most of the HIV/AIDS programmes in Ukraine. Currently, there are four grants with a total budget of US$141 million in Ukraine, which are managed by two local NGOs, one government organisation and one United Nations agency [93]. In 2015, the Ukrainian government agreed to take over most of the responsibilities as international donors planned to reduce funding. In 2018, Ukraine aims to cover 20% of all activities described in the National Strategy to Fight HIV/AIDS, rising to 50% in 2019 and 80% in 2020.

In addition, we observed that the number of HIV testing teams has diminished over the years as a direct consequence of reduced international funding. According to the national testing policies, an HIV testing team (both in-facility and outside medical facilities) should include a doctor, a nurse and, if possible, a peer-to-peer consultant or social worker (order No. 415, 2005). However, in practice, only a doctor or a nurse is performing HIV testing because of the limited number of professionals trained and willing to work with sex workers or drug users. Since 2015, there have been no international funds allocated to cover costs of health professionals in outreach testing activities among key affected populations in Ukraine, although it was proposed that the government would start funding them. Consequently, with no additional funds disbursed, the vast majority of health professionals stayed in general health facilities and do not participate in outreach HIV testing (e.g. in the NGOs, in the mobile clinic). Therefore, outreach HIV testing among FSWs is now commonly provided by a social worker; all positive test results should be followed by a confirmatory test in a health facility. Still, the stigma as well as the separation of AIDS clinics from other health facilities, which we address below, might impede access to public health services, including confirmatory testing.

Moreover, successive advocacy campaigns have by and large focused on treatment (accessible antiretroviral therapy); those efforts achieved their goal, as now antiretroviral therapy is fully funded from the Ukrainian national budget.

#### Vertical approach

In the early 1990s, as a result of the National HIV/AIDS Programme 1992–1994, a separate network of AIDS clinics spanning the country was established in order to address the ‘poorly researched disease’. The system put in place for HIV prevention and treatment has been a vertical rather than an integrated approach. This approach was driven mostly by fear and stigma towards ‘virus carriers’, those ‘suspected to have HIV’, ‘AIDS criminals’ and ‘AIDS stagnant’. Thus, people living with HIV were in a way excluded from general health practice and separated from all other patients. Later on, such separation was claimed to be an effective approach to overcome stigma towards people living with HIV in the general healthcare practice and to address the specific needs of people living with HIV. Over the years, this approach was institutionalised by various laws and orders, and so inadvertently aided in perpetuating inequality in accessing general healthcare. Many respondents from all sides of the programming landscape mentioned that this could further distance people living with HIV from general healthcare practice:

“*…some doctors say* ‘*We are not paid extra for work with HIV positive, go to your AIDS centre, there they receive +60% extra’. This is the way…this is the attitude at the polyclinics, it is very superficial… it is not normal…because they are not paid extras. But from other hand why to pay extras at all? If we all are equal, yes? And if potentially all the patients are infected… they supposed to…. despite of age, if this is granny or grandpa who came …potentially….eh to treat them as…eh…potentially dangerous…eh…to protect themselves…to use gloves…and all other. And not when they know that this is HIV-positive person who came… then, they put a space suit to take blood from a figure …. (sigh).*” (P01, NGO)

There have been a number of recent efforts to integrate HIV services into the general healthcare system (e.g. joint country HIV/tuberculosis programme supported by the Global Fund), which seem to be especially important in light of ongoing healthcare reform in Ukraine and the anticipated reduction in international funding.

#### Fragmentation

During our fieldwork, we observed how local NGOs funded by international donors and guided by WHO recommendations [94] implement self-testing or assisted testing (when a social worker provides pre- and post-test consultations and instruction on how to apply rapid HIV tests) among FSWs in Ukraine. In addition, much of the conversation in interviews centred on issues of lack of continuity of care and the challenge of integrating the HIV testing programmes with medical services and other types of care in the HIV landscape. Social and psychological services were mentioned as important aspects of prevention and access to testing by FSWs and those who work closely with them.

The limited cohesion of these services in most, but not all, NGOs and separation of these services from state clinics is seen as a significant barrier. This quote from a local researcher illustrates the issue of medical services within the health system and the placement of populations that are most vulnerable to HIV outside more socially acceptable forms of care (interviewer comments in bold):

“*There are services that provide this HIV testing, you can do it with an organisation called the HIV centre.”*

***That’s the government organisation?***

*Yes, this is a very Soviet type of organisation, to be honest. It was created in the late*

*Soviet times. Now they are created all over Ukraine, at least in big regional cities. It’s part of the Ministry of Health. Despite their name that they’re services to combat and prevent HIV, they’re focused only on medical problems – on testing and provision of ARV* [antiretroviral] *treatment, HAART* [highly active antiretroviral therapy] *treatment. There are no social services. And they exist separately. They were created in separate settings. And it also creates this problem you have of separate organisations. You’re separated from all others.* ***Separated from, say, primary care and the health system?***

*“Separated from primary care and all the health system*.” (P09, NGO)

#### Lack of coordination

At the organisational level, there is no central governmental coordinating body for programming in the entire country. In some instances, oblast-level governments are left to formulate their own policies and programmes, assuming they have the political will to do so. Fragmentation of communication and general service delivery means that coordination between civil society and the government is not as effective as it could be, were it fully functional and integrated. International organisations may have their own priorities and opportunities for implementing their own policies and programmes, but when government support is brought into question, it is less obvious how the Ministry of Health may take on a greater role in funding in the future. This is especially relevant, as all interviewees had some doubt as to the ability of the government to provide a robust response to the HIV situation in Ukraine after international donors leave the country.

A representative of one international donor agency suggested that HIV programming, including testing and treatment services, suffers from poor national governance, currently and in the past. As donor organisations attempt to transfer ownership of HIV programming to the government, both financially and organisationally, there is uncertainty about how programmes will be affected by the government’s potential failure to provide an adequate response to its new responsibilities. The manager of a public health NGO in Ukraine spoke about the organisation’s relationship with the government, especially with regard to the influence of donors like the Global Fund.

“*…we got this kind of freedom to do a lot of very effective programmes on the ground. The minus of this is that we don’t really agree about everything with the Ministry of Health. That’s why we don’t have a lot of political support. But we don’t have a lot of political opposition either because the money’s coming from the Global Fund. This is not governmental funding. So, this is the good thing. The bad thing is that, basically, if we need to get some money from government in the future, we need to be more engaged in the policy processes because we need to have our interventions institutionalised and acknowledged by the government*.” (P06, NGO).

The vertically oriented, donor-supported system of HIV care has, to date, operated outside the direct purview of government. Therefore, a coordinated strategy for HIV testing, treatment and prevention has not taken place with the input and ownership of all stakeholders, including FSWs themselves. One former sex worker emphasised that the lack of a harmonised response from both government and civil society has resulted in the challenge of navigating a disjointed health system that is not set up with the beneficiary in mind:

“*There is no unique... let’s say in one place all the services at once... client-centred approach, it doesn’t exist*.” (P01, NGO)

#### Separation of key affected populations

Separation of key affected populations from the rest of the health system is a result of the perpetuation of a vertical HIV system of care, which first took root in the 1992–1994 National HIV/AIDS Strategy. The issues of integration versus fragmentation were a huge part of what this local researcher stressed as part of the problem of the landscape.

“*You always push people to some other services, and they also created this maternal service for HIV-positive women as a separate entity, for example, in Kiev. Or as a separate ward in some regional centres. So, and it’s created from big heart, we wanted to have special services, but you discriminate because you separate people. You send them to these services. So, you can go to this strange system called HIV centre and have testing there. You may have testing at so-called anonymous cabinets. It’s also run by health administrations, but as a rule, they sit separately. It might be in primary care, or it might just be a cabinet, a room somewhere in municipal services.*” (P10, researcher)

When services for HIV are located outside normal primary care facilities, yet another category is placed on individuals who are trying to navigate this system, further stigmatising their status as FSWs or people who inject drugs and of HIV in general. Again, we see the walling off, or separating, of certain individuals from the rest of society, a practice, as the researcher mentioned above, reminiscent of Soviet-style public health management.

## Discussion

In contrast with many high-income countries [95], Ukraine, as a lower-middle income country [96], does not have government organisations dedicated to producing evidence-based policies, recommendations and guidelines, although some NGOs try to contribute (e.g. Alliance for Public Health and AIDS Foundation East West, etc.). To our knowledge, this is the first attempt to assess applied research, knowledge translation, knowledge exchange or evidence use with regard to HIV testing policies among FSWs in Ukraine. Overall, HIV testing policies are formed and implemented in the post-Soviet context through the vertical system of AIDS clinics, resulting in separation of key affected populations from the rest of the health system. Lack of government funding for the prevention of HIV created a gap into which international donors and local NGOs entered; however, their influential role had limited influence on the Ukrainian government to increase funding for prevention. Stigmatising and discriminatory forced and mandatory approaches to HIV testing have shifted towards voluntary testing, self-testing and assisted HIV testing, yet stigma remained a persistent barrier among FSWs to access testing. Fragmentation of services available to female sex workers, lack of coordination between civil society and the government sector, poor governance, ongoing reform of the health system and war might affect continuity and sustainability of HIV testing services – especially if and when the major donors significantly reduce their funding.

Overall, it seems that policies in Ukraine that were theorised and formulated over the years were indeed largely implemented as intended (e.g. forced and mandatory testing, creating a vertically oriented system of AIDS clinics and a strong focus on treatment). The lack of financial resources was filled by the international donors, which delivered stable funding, particularly for local NGOs from 2003. Thus, a vertically oriented, donor-supported, fragmented HIV system was created. Over the years, innovative HIV testing approaches among FSWs were driven by the priorities of international organisations and donors. The government sector was left behind and thus did not develop, as one respondent put it, “*a reflex to fund prevention*”. Even now, when it is expected that most of the HIV/AIDS programme will be funded by the government, there is a traditional reluctance to take over financial responsibilities regarding prevention. The notable lack of resources for prevention may threaten the sustainability of prevention programmes, which should be at the core of ongoing advocacy campaigns of local NGOs, as well as prevention, and its funding should be prioritised by international donors working in Ukraine.

In addition, the vertical, donor-supported HIV/AIDS health system may be effective in its direct activities – for example, Ghana has experienced some positive outcomes from Global Fund involvement in its HIV/AIDS system, but only with the right leadership and integration with the rest of its health system [97]. Conversely, a survey of francophone sub-Saharan African countries described how those with vertical health systems contributed to weakened general health systems and threatened responsible resource allocation [98]. In Mozambique, a health system made up of international donor programmes perpetuated inequalities in health programmes, staff shortages in public health services (a form of internal brain drain), and limited the control of local stakeholders over programme activities [99]. This fragmentation means that the vertical HIV/AIDS health system is effective in its direct activities, but the inadequacies of the larger health system are felt later down the line in health facilities. The government health system might therefore not be as effective, equal or responsive as it should be.

Furthermore, the present HIV/AIDS health system may also be partly explained by the crowding-out effect. As the Global Fund has provided predictable and stable funding since 2003, the Ukrainian government may have shifted its own funding to other sectors or might be reluctant to invest in a system that it could not sustain without international funding [100, 101], an issue that should be further investigated. We suggest this should be addressed in future research.

Moreover, Carroll et al. argued that, in Ukraine, evidence is managed within the framing of international donors and organisations working in the country, and that this evidence takes on meaning within political and social spheres [102]. This ‘data vacuuming’ or ‘audit’ culture by donors in health programming has been shown in other contexts to inhibit effective decision-making and outcomes [103, 104]. This may change if FSWs achieve what they see as the best solution for their problems with existing programmes, that is, asserting their voice and own ‘rationalities’ into programme evaluation and the policy-making process.

Similar to other post-Soviet countries, Ukraine faces persistent stigma towards key affected populations [105–108], which also likely contributed to government reluctance to fund prevention. The parallel structure of AIDS clinics, initially created in the early 1990s to overcome HIV-associated stigma, may have done more to produce the unintended consequence of further stigmatisation and distancing of people living with HIV from the more integrated primary healthcare system. This may be considered as a manifestation of persistent sex-work and HIV-related stigmas in the country, suggesting that the government, by creating a separate parallel system of AIDS clinics, revealed its social dominance over the disadvantaged ‘others’ [89]. Even though our results showed a shift from discriminatory forced and mandatory HIV testing to voluntary testing and even self-testing and assisted testing, prohibitive administrative (Article 181/1) [109] and criminal (Articles 130, 302, 303) [110] laws may impede access to healthcare services, including HIV testing. Despite activists’ attempts from organisations led by sex workers and other NGOs to introduce the first non-discriminatory evidence-based sex worker policies, aiming to tackle the negative side-effects of sex work prohibition (e.g. stigma and discrimination, social exclusion, unsafe working conditions, poor occupational health, low self-esteem, restriction on housing, travel, and parenting) [111–113], these have been unsuccessful because of lack of government and public support. At present, Ukraine is still, in many ways, in transition; its society lives with the memory of Soviet forms of control through infrastructural organisation. This includes all spheres of life, including the health system, which fully relies on its Soviet predecessor and, accordingly, employs and reinforces its values and norms. Recently announced healthcare reform (law No. 2168 – VIII, December, 2017) introduces even more uncertainties, and even though such reforms are very needed, the ongoing war in parts of the country might constrain their implementation. Moreover, without unified national guidelines, assisted or self-testing approaches could be differently interpreted and introduced by lay providers and thus people who test positive for HIV may not be linked to healthcare facilities for conformation tests, treatment and care. We do not know how and whether sex workers or their clients who tested negative would behave as perceptions of their own risk might decline, leading to less safe sex. Sex workers might also consider self-testing as a reason to skip regular health checks at health facilities, where they may face stigma and discrimination.

Also, as shown previously, “*societies in change*” might be more susceptible to the rapid spread of HIV [5]. In fact, a recent study emphasises this possible threat by showing how war has fuelled the spread of HIV in Ukraine [114].

This study did not specifically examine HIV testing among all key populations (e.g. men who have sex with men and people who inject drugs), yet many of the described forms of stigma and discrimination likely apply to these groups as well. We have noted this above, when possible.

### Methodological considerations

When designing this study, we aimed to incorporate methodological recommendations [115, 116] that include characterising the policy process as a whole and not focusing on single elements, incorporating descriptive contextualisation by using a historical approach and discourse analysis, and including ‘insiders’ (AT, KS) and ‘outsiders’ (JO, DE, JVL, JEWB) across all the stages of the research project. With regard to positionality, the ‘insider’ (AT) in this case worked in the international charitable fund Alliance for Public Health in Ukraine for 5 years, which enabled her access to stakeholders, and to contribute to the development of meaningful interview guide questions, and to deepen the understanding of the research context. The ‘outsider’ (JO), previously unfamiliar with the Ukrainian context, had a relatively unbiased natural curiosity in this setting, and thus was able to obtain fuller explanations from interviewees and recognise overarching themes that cut across a policy or the country.

Although we used the term ‘power’ on several occasions, we feel it is important to emphasise that we were rather limited in understanding where it lay and how it was exercised during the period 1992–2015. Moreover, our understanding of ‘power’ was restricted by policy documents as most were succinct, highly structured and written in officially accepted and politically neutral language (over the years, the language became more neutral). Therefore, we relied heavily on interviews as a more nuanced and comprehensive source of information regarding power dynamics in this case.

A greater number of interviews and larger diversity of interviewees would have enhanced the validity of the results. For example, most of the respondents were working in civil society. While many held different positions, had diverse backgrounds and drew on various sets of knowledge, the fact that many were situated in this sector may have provided a picture of the overall landscape of programming and policy-making that is skewed towards a civil society-focused interpretation. It is important to report that we did not receive any feedback from the Ministry of Health of Ukraine and National Police Office, despite having contacted them several times via different channels (e-mail and employees with whom we already had contact).

Furthermore, we interviewed three former sex workers, who represent a self-organised movement of sex workers in Ukraine. Including a larger diversity of sex workers (also from the rural areas) may have provided a broader view. We also only interviewed one participant to examine how war affects HIV testing policies. We were also unable to consider commercial actors and their role in policy formulation, or to examine how HIV testing policies were influenced by external forces.

Another possible limitation is the decision to conduct most interviews in English with non-native English speakers, which could decrease rapport and the ability to express ideas freely. All non-native English speaker stakeholders, when recruited, were asked whether they wished to use Ukrainian or Russian languages.

In addition, the reform of the health system was taking place, and FSWs were still attempting to find solidarity among themselves to mobilise as a community. It is possible, given all of the external circumstances mentioned, that some interviewees were reluctant to give candid answers. However, such influential events are ever-present in the country and are an integral part of understanding how the landscape functions. One would be hard pressed to find a time when extenuating circumstances, internal or external, are absent in Ukraine, especially in relation to HIV. This also suggests that the limitation of a participant group mainly drawn from civil society is, too, a finding – that even with repeated attempts to reach government bodies, they were unable or unwilling to speak to the researchers.

We strongly support further research that would precisely address how HIV policy formulation and implementation is influenced by complex cross-border, inter-organisational and network relationships [117–119], how it is influenced by in-country civil society and “*street-level bureaucracy*” [120, 121].

## Conclusion

Taking into account that Ukraine’s health system is still in transition, in which a new model is currently designed to replace the post-Soviet system, it is crucial to prioritise the sustainability of existing HIV testing services among female sex workers. Poor governance and fragmentation of the healthcare system might constrain continuity of HIV programming in the country. Moreover, reduced international funding, and persistent stigma and discrimination towards people living with HIV and sex workers, might impede the sustainability of HIV testing programmes. Yet, there is a window of opportunity for local CSOs to contribute to the development and implementation of HIV policies, especially taking into account the unique expertise of various organisations that for many years have served as key implementers of HIV testing campaigns and have the support of international donors.

Box 1 Stakeholder’s list (*n* = 19) Non-government organisations (NGOs) (*n* = 9)

 Deputy executive director, public health NGO

 Policy representative, public health NGO, former police officer

 Programme manager, public health NGO

 Project manager, international NGO

 Executive Director, NGO working with key affected populations

 Programme manager, NGO working in national HIV programming

 MD, who worked in state sexually transmitted infections clinic; Programme manager, public health NGO

 MD, Executive Director, public health NGO in Donetsk; trainer on HIV/AIDS prevention

 Policy officer for sex workers, public health NGO

Community representatives of sex workers (*n* = 3)

 Director of sex worker-led organisation, member of International network of Sex workers, civil society representative of European Economic region, former sex worker

 Head of the board of sex worker-led organisation, activist, former sex worker

 Manager of sex worker-led organisation, activist, former sex worker

Government organisations (*n* = 3)

 Doctor working in an HIV clinic

 Local researcher working in a state university

 Local researcher working in a state and in a private university

International organisations/donors (*n* = 4)

 United States doctor working for a United States agency

 Two representatives from an international health agency

 International donor representative

## Additional files

Additional file 1: Search terms: Database analysis of HIV testing policies among FSWs in Ukraine, 2006-2015 years. (DOCX 23 kb)

Additional file 2: Interview guide: Interviews with stakeholders, Kyiv, Ukraine, April-June, 2017. (DOCX 22 kb)

## Abbreviations

CSOs: civil society organisations
FSWs: female sex workers
NGO: non-governmental organisation
