# Supplementary material for: ‘Virus Carriers’ and HIV testing: navigating Ukraine’s HIV policies and programming for female sex workers
Source: Health Res Policy Syst. 2019 Feb 28;17:23. doi: 10.1186/s12961-019-0415-4 (PMC6394058; doi:10.1186/s12961-019-0415-4)
Supplement: Supplementary file 2 — Interview guide: Interviews with stakeholders, Kyiv, Ukraine, April-June, 2017. (DOCX 22 kb) [file 12961_2019_415_MOESM2_ESM.docx]

***Annex 2*** Interview guide: Interviews with stakeholders, Kyiv, Ukraine

**INFORMED CONSENT FORM FOR PARTICIPANT INTERVIEWS**

*Thank the participant for their time and introduce yourself.*

**Explain participant that:**

- You are being invited to take part in a research study, which has the objective of explicitly identifying which structural barriers for implementation are present with regard to HIV testing and access to care for female sex workers. We aim to gain insight on the connections between the policies that are made, the organizations that implement them, and the individuals that benefit (or not) from the implementation.
- It is important for us to hear about the different experiences, concerns and suggestions of different people and to learn about your experience with regard to HIV testing policies and programming.
- While we aim to help the community to promote HIV testing uptake among Ukrainian female sex workers, we cannot guarantee that we will be able to cover all of the needs of participants that may be identified during this study.

Do you have any questions about what I have just explained?

**Informed Consent:**

The interview will take approximately one hour. You will not receive any direct benefit from taking part in this study, however we hope that the information that you share with us will help to make HIV testing more accessible to female sex workers. We would like to record digitally our conversation. Only our researchers will hear or have access to the recording. We will keep all data safely in an office computer.

Do I have permission to record our conversation?

*Turn on the tape recorder if permission is given.*

In this interview I am going to ask you about your experience with regard to HIV testing policies and programming. Some of the questions may contain sensitive subject matter, and there is no need to share if you are uncomfortable. You are free to stop the interview at any time or to refuse answering some question.

Do you agree to participate in the study and to continue the interview?

**Consent Form** (to be filled ONLY if the interview is not recorded)

In this interview we will talk about your experience with regard to HIV testing policies and programming for female sex workers.

The interview will take approximately one hour. It will be kept confidential and anonymous within this study. You may use any nick-name you like. We will not record your name anywhere. In this interview I am going to ask you about your experience with HIV testing policies and programming for female sex workers. You are free to stop the interview at any time or to refuse answering some question.

Do you have any questions about what I have just explained?

Do you agree to participate in the interview?

_________________________________ ___________________

Signature of participant Date

**Interview Guide**

Interview length: approximately one hour

**1)** **BACKGROUND AND SITUATING THE INDIVIDUAL**

- Can you briefly tell me who you are, including your background and current position?
  - Place of origin or age?/lived in Kiev for a long time?
  - Why did you start with this line of work?
- Can you describe your day to day routine?
  - With whom do you collaborate? How?
- How does your work involve HIV? female sex workers? [if they didn’t say so already]

**2) HIV PROJECTS**

- Can you tell me about HIV projects in Ukraine?
  - How were they implemented over time? Examples?
  - How are they managed? Examples?
  - How are they regulated [m&e]? Examples?
  - What do you think about these projects? Why?

**3) HIV testing projects and policies**

- Do you think it’s important for female sex workers to test? Why?
- How does HIV testing for female sex workers operate? Can you illustrate?
  - How can female sex workers access testing?
  - How can female sex workers find information about testing places and projects?
- What are the approaches? Can you illustrate? [NGO mobile, clinical, home kit]
  - How is informed consent obtained?
  - What happens before and after testing? [pre- and post-]
  - How are results communicated?
  - Do you see this changing in the future? Why?
- What are some of the policies/regulations that have to do with HIV testing? (the current ones)
  - How does it work?
  - How are they developed?
  - How are they introduced?
  - What do you think of them? Why?
  - What about policies at a (government level/organization level) [whichever one respondent did not mention]? Examples?

**4) Barriers**

- What are barriers to HIV testing for female sex workers in Ukraine? Why?
  - Do you need to have a registration?
  - Do you need to have a passport?
  - What about administrative fine/criminal repercussions for prostitution? Do you think they influence access to testing or testing with NGOs? Why?
  - What about young people? Under 18? 16? [passport] [street kids]
  - Do you need to pay for testing? [oop]
    - What about treatment? [access, cost]
  - What do you think about paid testing? [GF new service delivery model]
  - What do you think about stigma towards female sex workers? Why?
- How can these be addressed on an organizational level? Governmental level?

**5) Facilitators**

- What can motivate female sex workers to test?
  - What do you think about incentives?
  - Are there different approaches that encourage testing? Examples?
  - What can different organizations do to encourage testing? And how? [p2p, outreach, campaigns]
  - What can be done on the governmental level?

**6) Future**

- How do you see testing in female sex workers in the future?
  - What are the goals for testing in the future?
  - How can that be accomplished?
- What is the role of the government? NGOs?
- What about the future of policies?
  - How can those be accomplished?
  - What are some of the challenges to policy formulation? Implementation?
- What about funding?
  - I know that some organizations are now thinking about Global Fund monies. Is there anywhere else organizations like yours can expect funding?
  - What will happen after global fund?
    - How will policies be influenced by this?
    - How did Global Fund influence policies?

**7) Supplementary questions on war in Eastern Ukraine**

- Can you tell me about the situation in eastern Ukraine?
- Is it relevant for this subject? Why/why not?
  - Have things changed very much?
  - It seems like you feel (strongly/ambivalent) about that.
- It’s been reported that more people have migrated from the east to Kyiv and other cities, including sex workers. What does this mean for testing programs?
  - What about for other types of social services or parts of society?
- Do you see the situation changing in the future?

**8) Concluding the interview**

- Do you have anything else to add?
- Do you want to ask me any questions?
- Do you have any suggestions for someone else to interview, whom might add some needed insight to the project?
- Do I have your permission use the responses you’ve given me for the purposes of my study, which will remain confidential?

Thank you for your time!

***Annex 3. Coding framework***

**Theme 1.Context/Landscape**

- POST-SOVIET CULTURE/country “in transition”
  - Separation
  - Fragmentation
  - Ukrainian “cultural” factors
  - Human rights
    - Stigma
    - Discrimination
  - Morality and Sexuality
  - Health informational propaganda

**Theme 2. Actors**

- GOVERMENTAL STRUCTURES
  - Parliament/national government
  - Ministry of Health
  - Local governments
  - AIDS clinics
  - “Dovira” cabinets
  - Other facilities
- CIVIL SOSIETY (NGOs)
  - Alliance
  - All Ukrainian Network of people living with HIV
  - Other
  - Key populations
    - FSWs
    - Other key populations
    - Risk
- INTERNATIONAL DONORS
  - Global Fund
  - UNAIDS
  - USAID
  - CDC
  - Others

**Theme 3. Content**

- RESOURCES
  - Health care products
  - Human resources
  - Health system financing
- HEALTH SYSTEM ORGANIZARION
  - Norms and instruction
  - Quality control
  - Policies and protocols
  - Health system dynamics/process
    - Health system financing
    - Health system organisation
    - Medical services
    - Social services
    - Integration/fragmentation
- PROGRAMMATIC DETAILS
  - Barriers and facilitators
    - Barriers
      - Criminalisation
      - Stigma
    - Facilitators
  - HIV programming
    - Testing
    - Treatment
    - Prevention
    - Data/evidence
    - Other programming (Health informational propaganda)
  - Conflict
    - Conflict
    - IDPs
